# Leukoencephalopathy with intracranial calcifications and cysts in an adult: Case report and review of literature

**DOI:** 10.4103/0972-2327.74198

**Published:** 2010

**Authors:** K. Ummer, K. A. Salam, Mohan L. Noone, V. G. Pradeep Kumar, Neena Mampilly, S. Sivakumar

**Affiliations:** Department of Neurological Sciences, Baby Memorial Hospital, Calicut, Kerala, India; 1 Department of Pathology, Baby Memorial Hospital, Calicut, Kerala, India

**Keywords:** Cysts, intracranial calcifications, leukoencephalopathy

## Abstract

Leukoencephalopathy, intracranial calcifications, and cysts (LCC) is a very rare cerebral disorder, first described in 3 children in 1996. It has subsequently been reported in adults and children from Europe and America, but has not so far been reported from Asia. We report an adult patient with pathologically proven LCC from a tertiary care hospital in South India. He presented with features of ataxia and raised intracranial pressure. Magnetic resonance imaging of the brain showed multiple bilateral cerebral cystic lesions along with diffuse white matter lesions in the cerebral and cerebellar white matter, and computed tomography of brain showed multiple calcifications in the white matter and basal ganglia. A large right cerebellar cyst causing mass effect was surgically excised. Histopathologic features were consistent with earlier reports of LCC and showed Rosenthal fibers, angiomatous changes, and calcifications. Our report suggests that although it is rare, LCC has a global distribution.

## Introduction

Leukoencephalopathy, intracranial calcifications, and cysts (LCC) is a very rare cerebral disorder, first described in 3 children in 1996.[1] It has been later reported from around the world in children and adults, with onset up to 59 years.[2] The clinical presentation is insidious and variable. Typically, initial symptoms are of raised intracranial pressure, later followed by focal neurologic deficits. All the reported patients have a characteristic triad of calcification in the deep cerebral nuclei and white matter, diffuse leukoencephalopathy, and multiple cystic brain lesions on brain imaging. The histopathologic findings described include a peculiar angiopathy and abundant Rosenthal fibers.[3] All these features justify the designation of LCC as a distinct, although extremely rare, nosologic entity.

We report an adult case with clinical, radiologic, and pathologic features consistent with LCC from a tertiary care hospital in South India.

## Case Report

A 50-year-old man presented in June 2009 with headache of 4 months duration, progressive unsteadiness of gait since 1 month and recurrent vomiting since 1 week. Headache was holocranial, throbbing, almost continuous, and tended to disturb sleep. Cough worsened the headache. He never had diplopia or visual obscurations. He felt unsteady while walking, but gave no history of falls. He did not have tremors or difficulty in using the upper limbs.

He had a past history of seizures during childhood, associated with fever, which had not recurred so far. He worked as a vendor for herbal medications, and was continuing to do so until a month prior to the onset of symptoms.

Clinical examination showed early papilledema, normal eye movements, bilateral finger–nose and heel–knee incoordination as well as dysdiadochokinesia. He had a spastic-ataxic gait.

Complete blood count, sedimentation rate, liver and renal function tests, serum calcium, phosphate, alkaline phosphatase levels, chest radiograph, and abdominal ultrasonogram were within normal limits. Serological tests for HIV 1 and 2 were negative.

**Figure 1:**
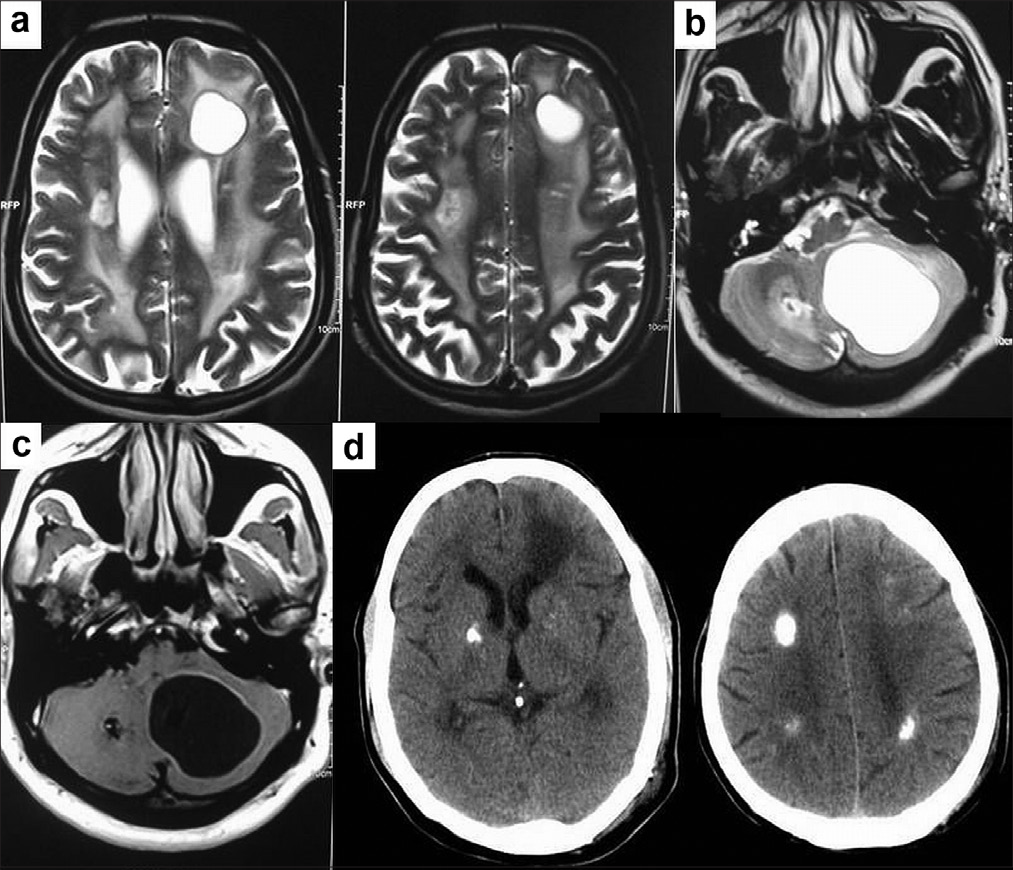
(a) T2-weighted (T2W) magnetic resonance imaging (MRI) of brain: bilateral cystic lesions and diffuse white matter lesions; (b) a large right cerebellar cyst compressing the 4th ventricle and brainstem; (c) T1W MRI brain after intravenous gadolinium enhancement of cyst wall; and (d) computed tomography of brain showing multiple calcifications

Magnetic resonance imaging (MRI) of the brain showed multiple bilateral cerebral cystic lesions and a large right cerebellar cyst with mass effect, compressing the 4th ventricle and brainstem. Diffuse T2 hyperintense lesions were seen bilaterally in the subcortical and cerebellar white matter. The cystic lesions enhanced with contrast administration. No hemorrhage was noted [Figures 1a–c].

Lumbar cerebrospinal fluid (CSF) was slightly xanthochromic and showed 26 leukocytes (45% polymorphs, 55% lymphocytes) with protein of 106 mg% and glucose of 149 mg%. CSF cryptococcal antigen was negative.

The differential diagnosis considered included cystic metastases, infective cysts, and tumefactive demyelination. We decided to proceed with posterior fossa craniectomy and excision of the right cerebellar cyst. Peroperatively, the cyst contained brownish fluid and showed visible calcification in the wall.

**Figure 2:**
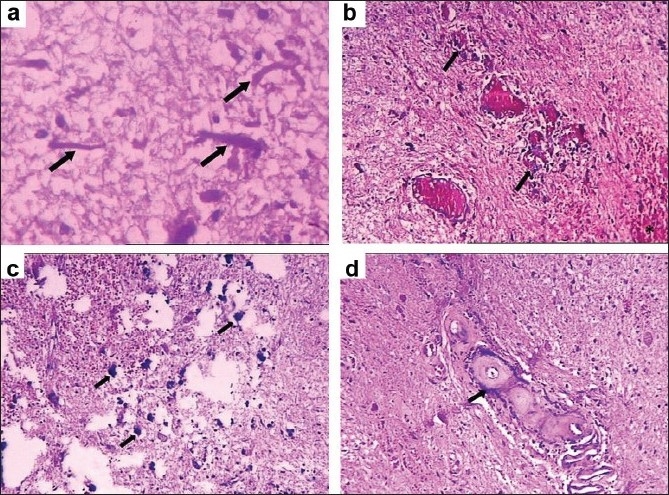
Photomicrograph of the biopsy showing (a) intensive gliosis with Rosenthal fibers (arrows); (b) angiomatous changes (arrows) with microhemorrhages (open arrow); (c) microcalcifications (arrows); and (d) concentric calcification around blood vessels (arrow). Hematoxylin and Eosin, a: ×400, b–d: ×100

A computed tomography of brain was done postoperatively and confirmed the presence of multiple calcifications in the white matter and basal ganglia [Figure 1d]. Histopathology of the excised cyst wall showed intensive gliosis with Rosenthal fibers, prominent angiomatous changes, microcalcifications, and microhemorrhages. Angiomatous changes consisted of numerous small blood filled vessels with many showing hyalinized walls. Gliosis was pilocytic and showed plenty of Rosenthal fibers. Microcalcifications were extensive and there was also concentric fine calcification around blood vessel walls. Foci of microhemorrhages and hemosiderin pigment deposits were also noted in relation to the abnormal blood vessels [Figures 2a–d].

Postoperatively the patient reported improvement in headache, while ataxia persisted. He was discharged on the 8th postoperative day and is being followed-up.

## Discussion

We report an adult patient diagnosed with LCC from South India. Our patient has all the characteristic clinical, radiologic, and pathologic features of LCC described in the previous reports.

Our patient presented with raised intracranial pressure and cyst-related mass effects, which are the 2 main presenting features of LCC. Large posterior fossa cysts causing pressure effects requiring surgical decompression are frequently noted and may be considered as a characteristic feature.[4] Some of the patients tend to be relatively well preserved, with no major cognitive or motor deficits, until they develop symptoms related to the mass effect of large cysts.[5] Repeated surgical procedures may be required to relieve symptoms caused by the enlarging cysts.

Some patients diagnosed with LCC also have Coat’s retinopathy.[3] It has been proposed that Coat’s retinopathy is a part of the spectrum of this disorder–the combined presentation has been termed as “cerebroretinal microangiopathy with calcification and cysts” or Coat’s plus.[6] However, our patient did not have any evidence of retinal involvement.

Raised CSF protein has been described in a patient diagnosed with LCC and large posterior fossa cysts.[1] Our patient also had raised CSF protein, but also had pleocytosis, which has not been previously described, and is of unclear significance–the possibility of an associated sterile meningeal inflammation may be considered.

Histopathology of LCC is characterized by angiopathy, calcification, and Rosenthal fibers, and the tissue from our patient showed all these cardinal features. The primary abnormality is probably the obliterative cerebral angiopathy, which involves small vessels. Dystrophic calcifications (via slow necrosis) and formation of cysts and secondary white matter abnormalities may be secondary changes.[1] It has also been suggested based on MRI characteristics that the white mater hyperintensities on T2-weighted images represent vasogenic edema affecting the parenchyma.[3]

In the first reported adult case, genetic analyses did not identify any significant mutations in 2 candidate genes, glial fibrillary acidic protein (GFAP) and cerebral cavernous malformation (CCM), although a mutation of unknown importance was identified in the gene for GFAP.[7]

The cause of this mysterious disorder remains unknown, but is probably genetic. Further genetic testing may provide more insights. The other case reports are from France,[1] Switzerland,[2] Brazil,[3] Turkey,[4] the United States,[5] the United Kingdom,[6] and Finland.[8] We add India to this list, raising the possibility that although rare, LCC has a global distribution.
